# GIS based method for mapping actual LULC by combining seasonal LULCs

**DOI:** 10.1016/j.mex.2023.102472

**Published:** 2023-11-04

**Authors:** Md. Sharafat Chowdhury

**Affiliations:** a Department of Geography and Environment, Jahangirnagar University, Dhaka, Bangladesh; b Centre for Remote Sensing and GIScience, Dhaka, Bangladesh

**Keywords:** Geographic information systems, Land use and land cover classification, Seasonality, Overlay, Actual LULC, Actual LULC modelling by incorporating seasonality effect

## Abstract

One of the most significant applications of remote sensing data is to prepare land use and land cover (LULC) maps. LULC maps are always affected by seasonality and a single LULC map of a particular month is prepared to represent a year in most of the research, especially in change detection research. This does not represent the real view of the landscape because the seasonal variation of different LULC types is always overlooked. Considering the issue, the current method aims to solve the problem by incorporating seasonal LULC using the raster overlay method to remove the seasonality effect on LULC classification. To apply this method, a minimum of two seasonal LULC maps is required for a single study year. The map needs to overlay and then reclassify according to the stable and rotational LULC pattern of the study area. This method will replicate the actual LULC pattern of a study area from satellite images. Summary of the method is as follows:

• LULC of each season was classified using image classification technique.
• LULC of each seasons are coded and combined using overlay technique.
• Combined map is reclassified to prepare the actual LULC map.

## Graphical abstract

**Figure:**
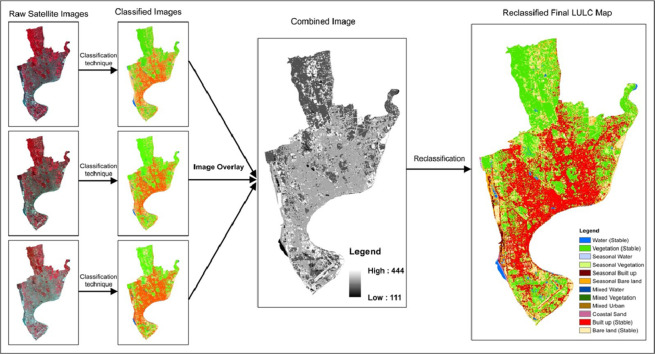

Specifications Table

**Table:** 

Subject area:	Environmental Science
More specific subject area:	GIS and Remote Sensing Application
Name of your method:	Actual LULC modelling by incorporating seasonality effect
Name and reference of original method:	NA
Resource availability:	Equipment: Seasonal satellite images and image classification toolset Data: Open source Landsat images are available at USGS earth explorer (https://earthexplorer.usgs.gov/) Processed Data will be made available on request. Software: AcrMap 10.5 Hardware requirement for software: CPU Speed 2.2 GHz minimum; Hyper-threading (HHT) or Multi-core recommended; Memory/RAM minimum 4 GB, recommended 8 GB; Disk space minimum: 4 GB recommended: 6 GB or higher; Display properties 24-bit colour depth

## Method details

Seasonality significantly affects the accuracy of LULC maps extracted from satellite imageries. The effect of seasonality on LULC maps has been studied and emphasis was given to comparing and improving the accuracy of the classified maps [1], [2], [3]. Spectral-temporal mixing of cloud-free satellite images was also been applied to reduce the seasonality effect and to improve map accuracy [[4], [5]]. Another emphasis has been given to selecting the best season to delineate LULC of a particular environmental condition or extracting a particular feature [[5], [6]].

LULC has significant application in several scientific studies [7,8] such as LULC change analysis [9], LST analysis and its impact analysis [10], hazard assessment [11,12], climate change and impact analysis [13,14], large-scale environmental monitoring [15,16] and environmental degradation assessment [17,18] etc. Besides, LULC and LULC change maps are the major input variables of many models such as climate change models [19], [20], [21], hazard assessment models [22] and hydrological models [23], [24], [25]. The accurate LULC helps take proper action for sustainable environmental management, hazard mitigation and climate change policy development [7,8]. LULC plays a key role in the sustainable management and development of urban and rural areas, agricultural land, watersheds or wetlands, river banks, forests etc. [2].

However, preparing an accurate LULC always depends on the quality of the satellite image. Cloud-free satellite image provides more accurate LULC of a region. Researchers always emphasized taking a cloud-free image and mapped LULC of a single season which is considered as the representative of a single year [6,9,26,27]. This type of classified map, though it has higher accuracy, is not appropriate for field application as some of the seasonally affected features (vegetation, water and bare land) are ignored in this type of classification. Due to the cloud effect, cloud-free satellite data may be available for two or three seasons for LULC classification. The seasonal change affects the extent of the water body and alters the pattern of vegetation cover which ultimately affects the extent of built-up areas and bare land. To get an accurate LULC map of a single year it needs to consider the seasonally varied landscape features such as seasonally altering water body, vegetation type and bare land characteristics. Change in the built-up areas within this short time is negligible and it can be considered a fixed amount.

In this method, seasonal variation of LULC is considered and from the combination of LULC of three seasons a final LULC map is prepared using ArcMap 10.5 software. This type of classification will produce an accurate LULC of a given year and will be very useful for scientific application. The method is discussed below.

## Data acquisition and source

Landsat 8 OLI data was acquired for three seasons of post-monsoon, winter and summer for the study area. In Bangladesh, cloud-free Landsat images are available from November to March. During April and May, though they are the months of the summer season, sometimes cloud affects the Landsat images. So, images of the three seasons are collected following the months and seasons shown in Table 1.

**Table 1:** Description of the acquired satellite image.

Seasons and Corresponding Months	Acquisition Date	Path and Row
Post-monsoon (October to November)	2021/11/19	136 and 45
Winter (December to February)	2022/01/08	136 and 45
Summer (March to May)	2022/03/21	136 and 45

The LULC is prepared in two steps: in the first step, LULC of three seasons are prepared and the LULCs are coded according to category. In the second step, the three maps are combined and reclassified according to the combination of the code.

### LULC classification for single season

A common, widely accepted and intensively used LULC classification method is the Maximum Likelihood classification method [28,29]. LULC of the three seasons are classified using the Maximum Likelihood classification technique. To classify the LULC maps, training samples are taken for individual images of each season.

**Fig. 1:**
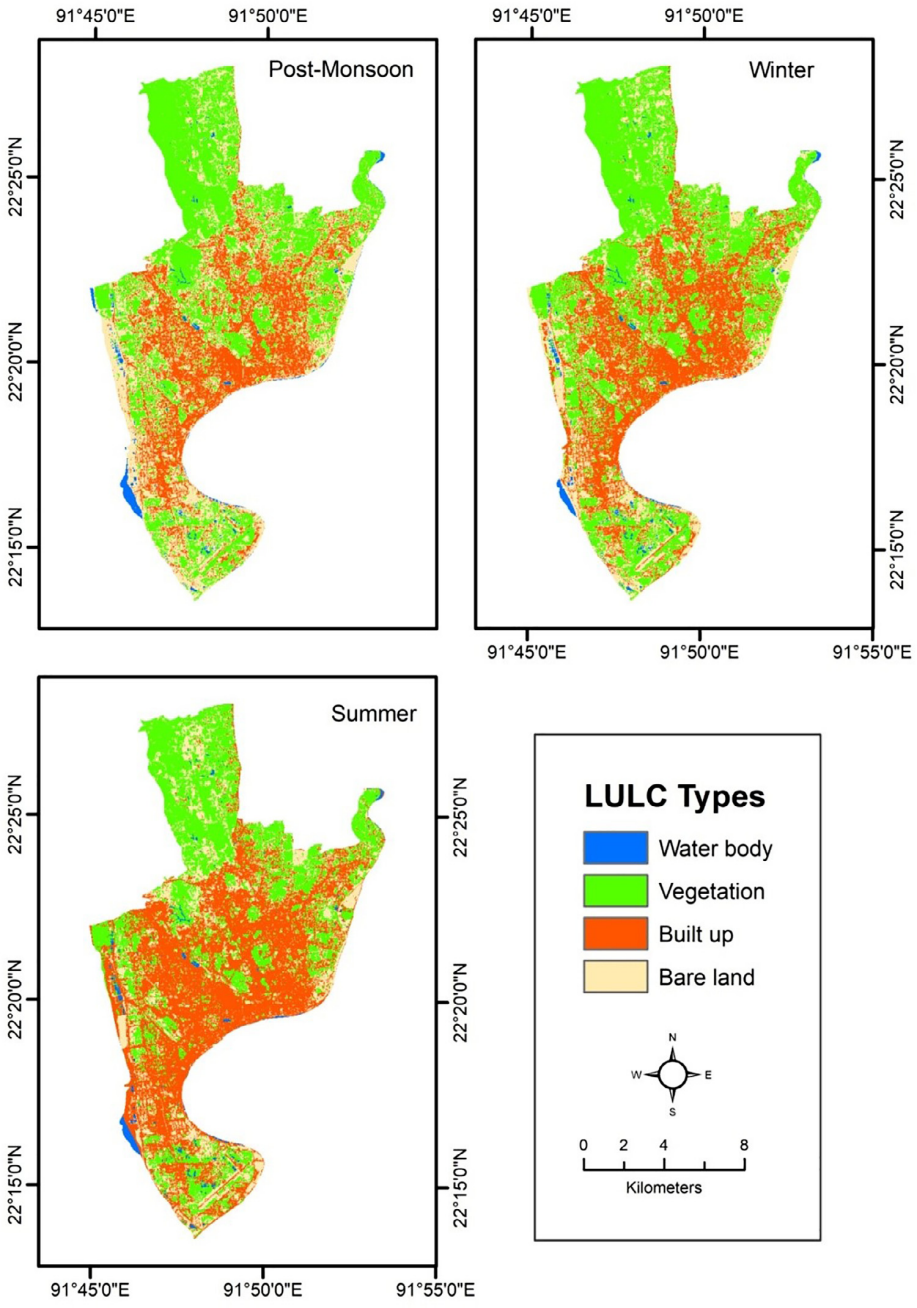
Seasonal land use and land cover map of the study area.

The classified maps are shown in Fig. 1 and the obtained accuracy of the maps is shown in Table 2. The graphical distribution of LULC maps shows that types of LULC vary according to the variation of the season (Fig. 1). The accuracy of the classified maps are measured using commonly used statistical indices of user accuracy, producer accuracy overall accuracy and kappa coefficient [30], [31], [32]. The accuracy (Overall Accuracy and Kappa Coefficient) of the LULC maps and the accuracy of the LULC types (User Accuracy and Producer Accuracy) of the LULC maps also vary according to the variation of the seasons (Table 2).

**Table 2:** Accuracy of the classified maps (PA = Producer Accuracy; UA = User Accuracy; OA = Overall Accuracy; Kc = Kappa Coefficient).

Season	Code	LULC Type	PA	UA	OA	Kc
Post-monsoon	1	Water body	1.00	0.92	0.96	0.95
	2	Vegetation	0.97	1.00		
	3	Built up	1.00	0.93		
	4	Bare land	0.89	1.00		
Winter	1	Water body	1.00	0.82	0.92	0.89
	2	Vegetation	0.88	0.87		
	3	Built up	1.00	1.00		
	4	Bare land	0.83	1.00		
Summer	1	Water body	1.00	0.92	0.94	0.92
	2	Vegetation	0.94	0.87		
	3	Built up	0.97	1.00		
	4	Bare land	0.86	0.98		

### Combining the LULC maps by simple overlay method

**Fig. 2:**
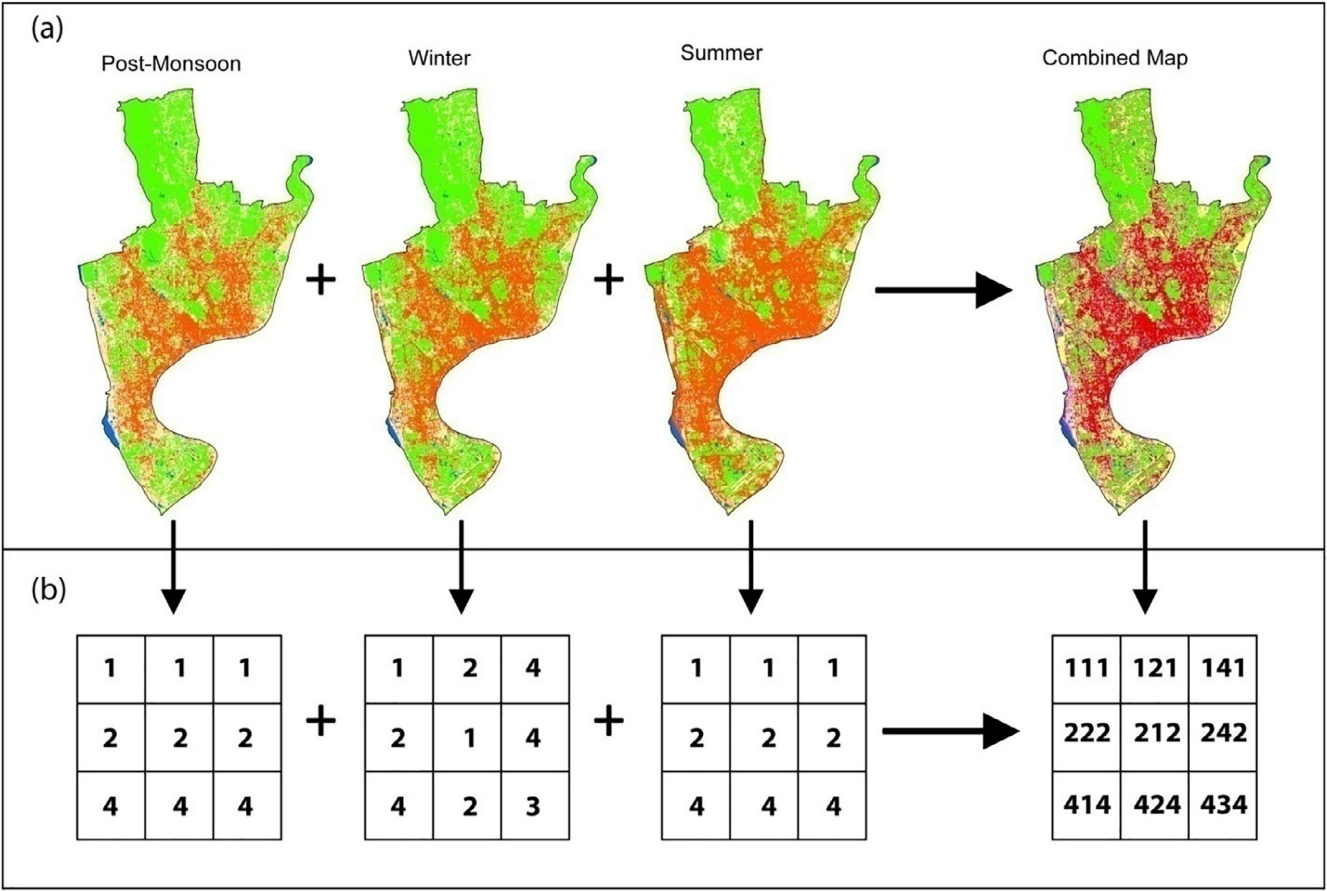
a) Overlay of the LULC maps of the three seasons, and b) pixel value of the final map derived from seasonal map overlay (Figure legend: 1=water, 2=vegetation, 3= built up and 4= bare land).

The LULC types of maps are coded to identify the changes or rotation of the LULC types around the seasons. Codes of the LULC categories are given in Table 1. Using a simple overlay technique of ArcMap 10.5 software the maps are combined into a single map. This type of technique also can be applied to detect the change in LULC between two years. In this case, three years are combined to observe the rotational LULC to demarcate and reclassify them (Fig. 2.a and b).

### Reclassification of combined map

**Fig. 3:**
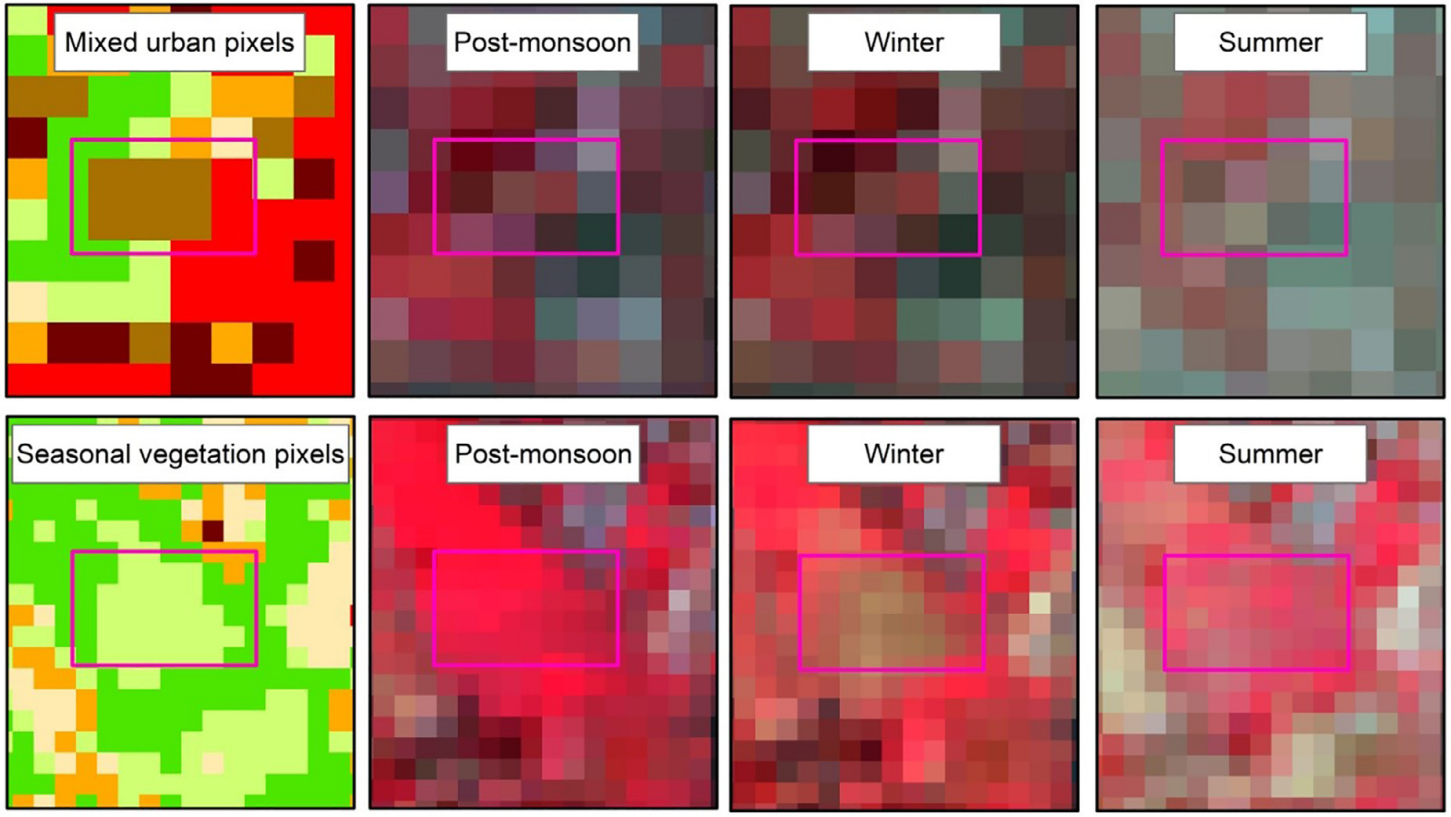
Reclassification of Mixed Urban and Seasonal Vegetation by visual interpretation.

After the combining the three maps, the combined map got three codes from the three seasons as shown in Table 3 and Fig. 2.a and b. The combined map is now reclassified following rotations of the codes. Such as when the code is 111 this means the area was water body in the three consecutive seasons. So this is considered as stable water body. When the code is 121 it is considered as seasonal water body. In this type of classification, maximum number of similar rotation is considered as final LULC class such as water rotated two times. Again, the code 212 is considered seasonal vegetation as in one season it drowned under water but most of the times dominated by Vegetation cover. When three different categories aroused, it is carefully interpreted visually from the three consecutive images and final decision was made (Fig. 3). The code 324 is considered as mixed urban as this feature is dominant in the urban areas and the spectral signature changes during summer identified it as bare land. The common assumption followed during visual interpretation was the association, proximity, colour depth and the mixture pattern of the class. A manual and the assumptions for visual interpretation are added in the Appendix. The visualization of the different codes and corresponding false colour combinations can also be found there.

**Table 3:** Reclassification of the combined map (1= Water body, 2= Vegetation, 3= Built up and 4= Bare land).

Post-Monsoon	Winter	Summer	Combined	Final LULC Type
1	1	1	111	Water (Stable)
2	2	2	222	Vegetation (Stable)
1	1	2	112	Seasonal Water Body
1	2	1	121	Seasonal Water Body
2	1	1	211	Seasonal Water Body
1	1	3	113	Seasonal Water Body
3	1	1	311	Seasonal Water Body
1	1	4	114	Seasonal Water Body
1	2	4	124	Seasonal Water Body
1	3	4	134	Seasonal Water Body
1	4	1	141	Seasonal Water Body
4	1	1	411	Seasonal Water Body
3	1	2	312	Seasonal Water Body
3	2	1	321	Seasonal Water Body
2	1	2	212	Seasonal Vegetation
2	2	1	221	Seasonal Vegetation
2	2	3	223	Seasonal Vegetation
2	3	2	232	Seasonal Vegetation
3	2	2	322	Seasonal Vegetation
1	2	2	122	Seasonal Vegetation
2	2	4	224	Seasonal Vegetation
2	3	4	234	Seasonal Vegetation
2	4	2	242	Seasonal Vegetation
4	2	2	422	Seasonal Vegetation
3	1	3	313	Seasonal Built Up
3	3	1	331	Seasonal Built Up
2	3	3	233	Seasonal Built Up
3	2	3	323	Seasonal Built Up
3	3	2	332	Seasonal Built Up
3	3	4	334	Seasonal Built Up
3	4	3	343	Seasonal Built Up
4	3	3	433	Seasonal Built Up
1	4	4	144	Seasonal Bare Land
4	1	4	414	Seasonal Bare Land
4	4	1	441	Seasonal Bare Land
2	4	4	244	Seasonal Bare Land
4	2	4	424	Seasonal Bare Land
4	4	2	442	Seasonal Bare Land
3	4	4	344	Seasonal Bare Land
4	3	4	434	Seasonal Bare Land
4	4	3	443	Seasonal Bare Land
2	1	4	214	Seasonal Water Body
3	4	1	341	Seasonal Water Body
4	1	2	412	Mixed Water
1	4	2	142	Mixed Water
2	4	1	241	Mixed Water
1	4	3	143	Mixed Water
4	1	3	413	Mixed Water
2	4	3	243	Mixed Vegetation
3	2	4	324	Mixed Urban
3	4	2	342	Mixed Urban
4	2	3	423	Mixed Urban
4	3	2	432	Mixed Urban
3	1	4	314	Coastal Sand
3	3	3	333	Built up (Stable)
4	4	4	444	Bare land (Stable)

### Prepared actual LULC map

**Fig. 4:**
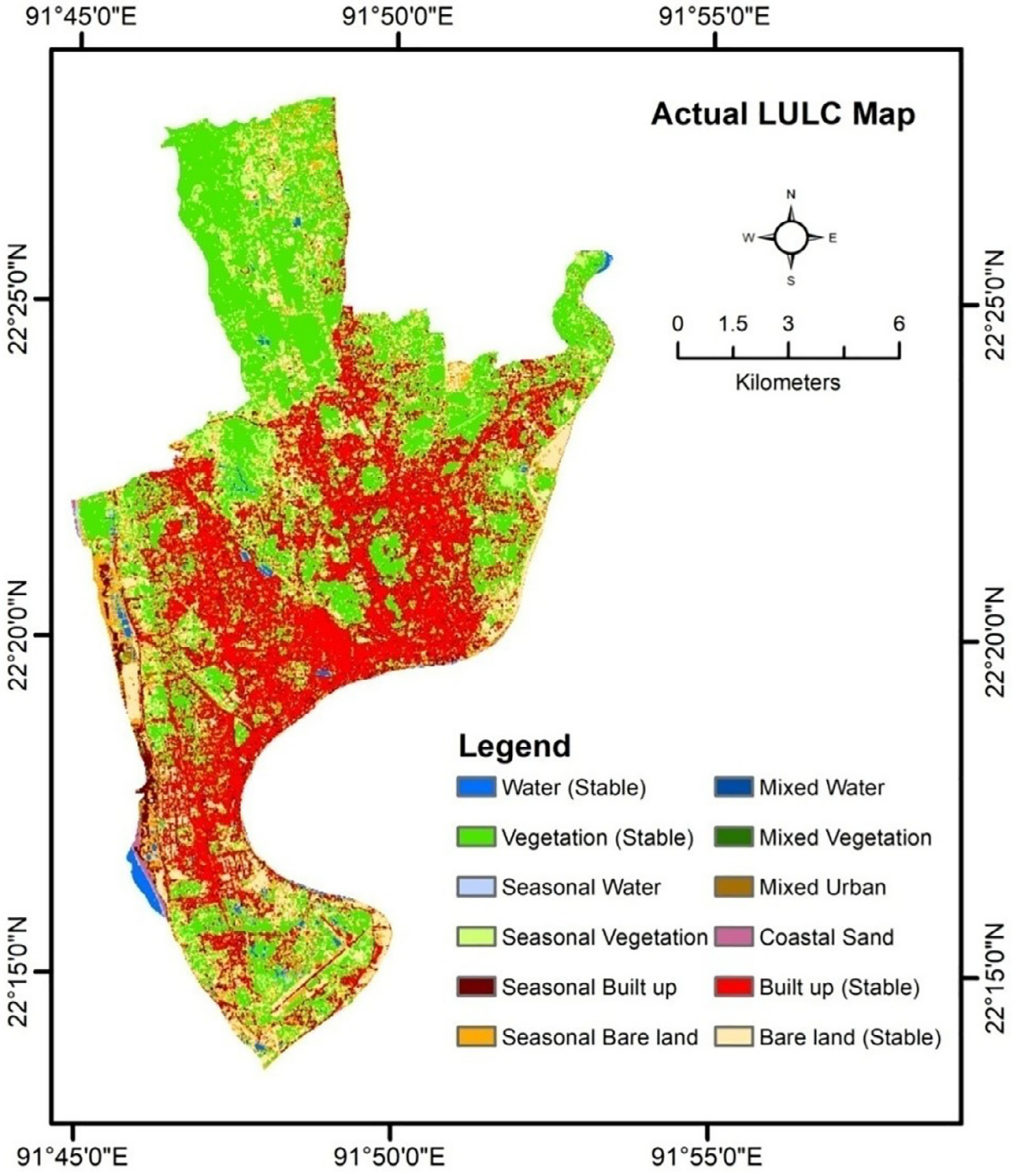
The final LULC map of the study area reclassified from the combined map.

The final map has twelve types of LULC namely Water (Stable), Seasonal Water, Seasonal Vegetation, Mixed Water, Seasonal Bare land, Vegetation (Stable), Seasonal Built-up, Mixed Vegetation, Coastal Sand, Mixed Urban, Built-up (Stable) and Bare land (Stable) (Fig. 4). The characteristics of LULC classes and the measured area are shown in Table 4.

**Table 4:** Characteristics of the final LULC types and the measured area of the LULC types.

LULC	Area	Characteristics
Water (Stable)	1.7	Perennial Water remains same types in all seasons
Seasonal Water	0.8	Changes in two seasons and finally returns to the previous class
Seasonal Vegetation	19.6	Changes in two seasons and finally returns to the previous class.
Mixed Water	0.14	Converts into three category around the seasons.
Seasonal Bare land	21.8	Changes in two seasons and finally returns to the previous class.
Vegetation (Stable)	50.0	Actual Vegetation Cover remains in all seasons
Seasonal Mixed Built up	13.9	Vegetation Cover exposed during another season. Bare land detected as built up in another season.
Mixed Vegetation	0.09	Converts into three category around the seasons but mostly vegetation dominant or located along vegetation cover.
Coastal Sand	0.5	Sands in the coast or in river bank, sometimes classified as built up areas
Mixed Urban	4.5	Converts into three categories around the seasons located in urban areas or along built up areas.
Built up (Stable)	40.0	Actual Built up area remains in all seasons
Bare land (Stable)	16.6	Actual Bare land remains in all seasons

### Merits of the proposed classification

• This type of classification provides pixel-based LULC maps.
• This classification process can be done in any GIS software which supports the raster overlay method or has a raster calculator.
• The method is simple to handle and no difficult mathematical expertise is required. Anyone who knows the LULC change detection method can easily understand and apply this method.
• Reduces the complexity of spectral-mixing of seasonal images or mixing of multi-sensor data
• Reduces classification time and cost.
• Produces actual LULC of a given year considering the dynamics of LULC
• The method will be very helpful for actual LULC change detection of a given area
• Provides actual LULC information on the study area which will be very helpful for policy or decision-making
• In agricultural areas, this classification will help to demarcate rotational agricultural land
• In forestry, it will help to demarcate the seasonal vegetation and its changing pattern over the season
• The phenology of LULC in any environmental condition can be detected using this method.

### Demerits/Limitations of the proposed classification

• Complexity is aroused during the reclassification of the mixed pixels and it requires visual interpretation using all the raw satellite images
• Sometimes it becomes tough to categorize the mixed pixels due to the similarity in the spectral signature of the neighbouring pixels.
• A clear knowledge of the study area is required
• Need to depend on the accuracy of each season's map.

### Further replication of the proposed classification

• Other LULC classification algorithms can be used to prepare a more accurate LULC map of each season such as random forest, KNN, ANN etc. this type of classification algorithm will produce better accuracy of the primary LULC maps which will ultimately increase the actual representation of the LULC pattern.
• At least two seasons should be considered to get the real LULC of the study area. The inclusion of more seasons will increase the reliability of the map. The inclusion of more seasons will provide more LULC classes which will be more representative of reality.
• The method can be used in other types of satellite data such as Landsat 7 EMT+, Landsat 4/5, sentinel, MODIS, ASTER etc. Similar satellite images of different seasons will produce better results. If a different satellite sensor is used, rescaling and re-sampling, and geo-referencing of the image should be done to make the pixels comparable.
• Application of machine or deep learning algorithm during preparation of each season's LULC map will subsequently increase the reliability and acceptability of the final map.

### Limitation of the current research

• Only one classification method of Maximum Likelihood is used for LULC preparation of a season.
• Some areas containing sand cover along the coast produce complexity in reclassification. They are classified as built-up in the summer season water bodies during the post-monsoon season and bare land during the winter season. This type of mixing also occurs in dense urban areas. Demarcation of this type of category produces errors.
• Only one urban area is classified considering the seasonal rotation of LULC but other environmental settings are not tested.

## Remarks

A simple and easy-to-handle method of actual LULC preparation from seasonal satellite data is presented. The classification method incorporates seasonal LULCs to prepare a final LULC map of a given study area where the seasonal rotational land cover of seasonal phenology of land cover is considered. Removing the seasonality effect, the method properly classifies the LULC of a given area. The method will be very helpful for scientific research of LULC mapping and change detection and other scientific research where the true representation of LULC is of significant importance. Though there are some limitations in the current research the method can be replicated for other environmental conditions. Any GIS software that supports raster overlay or raster calculator can be used to replicate the method. Satellite data from other sensors can also be engaged to classify actual LULC maps.

## Ethics statements

Not Applicable

## CRediT authorship contribution statement

**Md. Sharafat Chowdhury:** Conceptualization, Methodology, Software, Visualization, Writing – original draft, Writing – review & editing.

## Declaration of Competing Interest

The authors declare that they have no known competing financial interests or personal relationships that could have appeared to influence the work reported in this paper.

## Data Availability

Data will be made available on request.

## Appendix Supplementary materials

Image, application 1
