# Supplementary material for: GIS based method for mapping actual LULC by combining seasonal LULCs
Source: MethodsX. 2023 Nov 4;11:102472. doi: 10.1016/j.mex.2023.102472 (PMC10652103; doi:10.1016/j.mex.2023.102472)

**Assumption of Mixed Class Reclassification**

The basic assumptions of mixed class classification are:

- The combination of built-up with vegetation and bare land is considered mixed built-up. Commonly, the presence of built-up in one season and two different classes in the other two seasons is mixed built-up. Due to the presence of a roof-top garden and the close association of vegetation and impervious surface, the signature of built-up areas has a high chance of altering into vegetation or bare surface.
- The combination of water in any other two classes of two different seasons is considered mixed water. The presence of water in one season and two different classes in the other two seasons is considered mixed water. Water bodies can reflect different spectral signatures due to water depth and the growth of seasonal floating vegetation which can alter reflectance and sometimes it is classified as built-up, bare land or vegetation.
- For mixed vegetation classification emphasis was given to pixel texture.

To increase the reliability of the mixed class classification visual interpretation will be very helpful.

**Criteria to be Followed in Visual Interpretation**

For mixed urban classification association with other LULC classes should be followed. In the case of urban vegetation, bare land has a high chance of being mixed up. There is a very low chance of water pixel mixing in an urban setting. In the case of water body, vegetation and bare land has high chances of mixing and there is little chance of built-up area mixing. Again the association of the mixed class should be observed, and proximity to other LULC classes also needs to be considered. Such as in Annex 3. d, it is a pond which is covered by light vegetation during winter; in summer, pixel colour becomes faded. The variations of the pixel depth during the seasons also need to be considered. Such as in Annex 2. c, the built-up pixels showed three different depths in three seasons. During post-monsoon season, the proximity of vegetation cover increased the depth of red in false colour combinations. In conclusion, the following criteria needed to be followed for visual interpretation:

- Types of land use/cover in the study area
- Association of mixed pixel
- Variation of colour depth in false colour combination in different seasons
- Probability of class mixing in the study area such as in the study area there is a low chance of water and built-up area mixing
- Patterns of mixing over the seasons such as if built-up and barren lands found in two seasons there is a high probability of becoming mixed urban of that pixel.
- For mixed water classification quick decision can be taken based on one water pixel during dry seasons such as if the pixel is water during winter or summer and the other classes in other two seasons the pixel is mixed water.

Annex 1: Mixed Vegetation Classification (2= Vegetation and 3=Urban and 4= Bare land); false color combination is band 5, 4 and 3


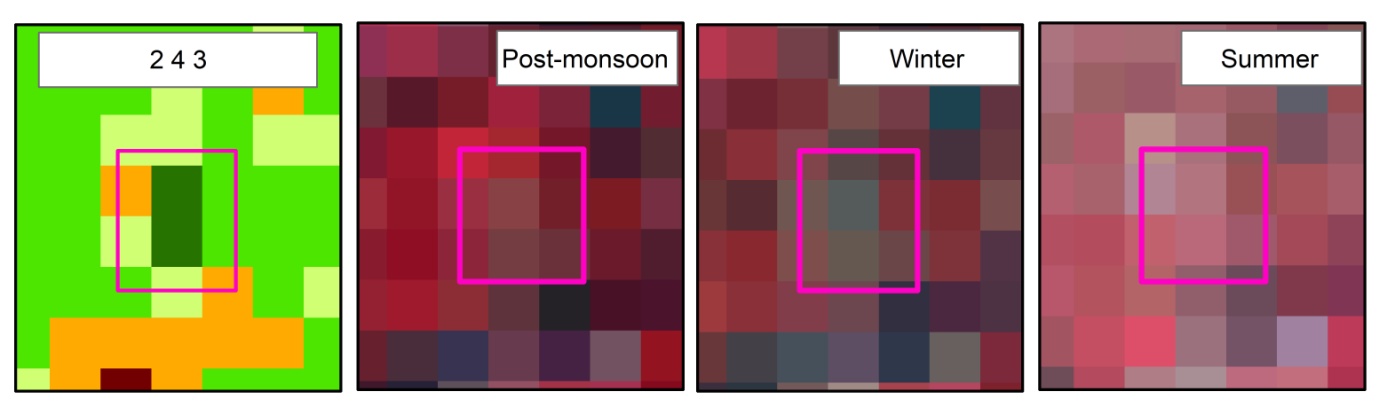


Annex 2: Mixed Urban Classification (2= Vegetation and 3=Urban and 4= Bare land); false color combination is band 5, 4 and 3


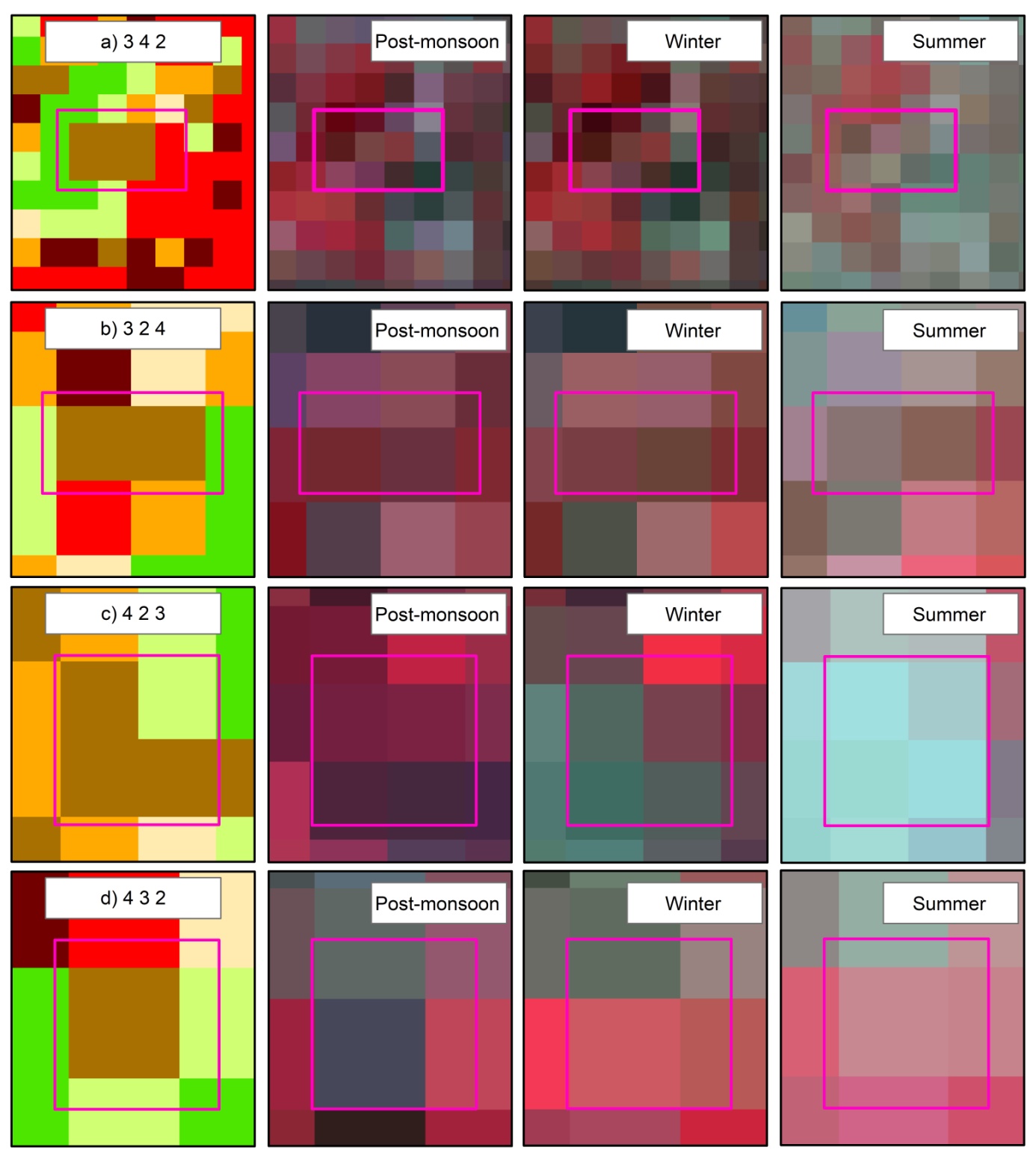


Annex 3: Mixed Water Classification (1 = Water, 2= Vegetation and 3=Urban and 4= Bare land); false color combination is band 5, 4 and 3


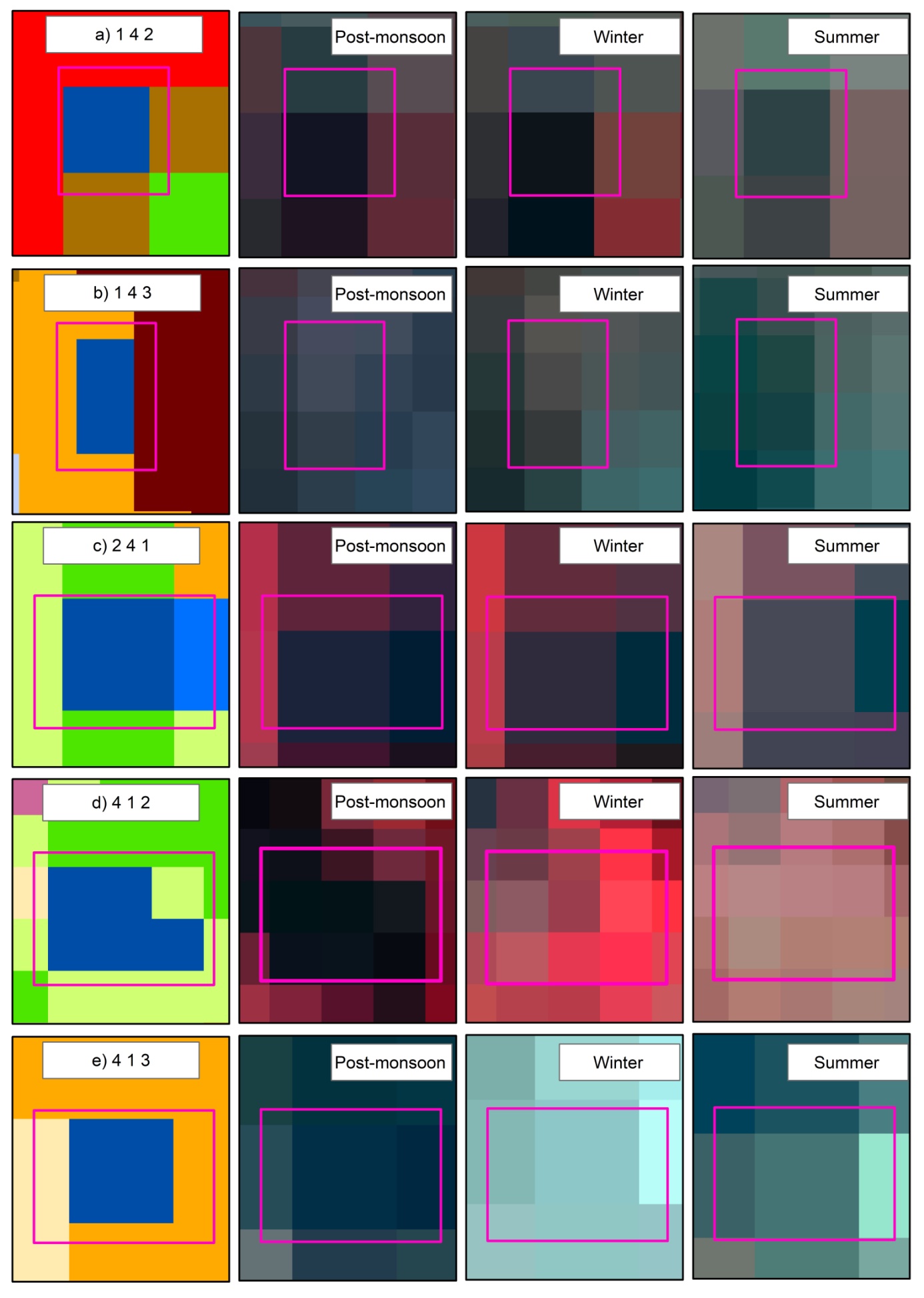

Supplement: Supplementary file 1 [file mmc1.docx]
